# Trinity of Three-Dimensional (3D) Scaffold, Vibration, and 3D Printing on Cell Culture Application: A Systematic Review and Indicating Future Direction

**DOI:** 10.3390/bioengineering5030057

**Published:** 2018-07-23

**Authors:** Haobo Yuan, Ke Xing, Hung-Yao Hsu

**Affiliations:** School of Engineering, University of South Australia; Mawson Lakes Blvd, Mawson Lakes 5095, Australia; Ke.Xing@unisa.edu.au (K.X.); Hung-Yao.Hsu@unisa.edu.au (H.-Y.H.)

**Keywords:** cell culture, 3D scaffold, dynamicity and dimensionality, dynamic scaffold, 3D static or passive scaffold, future scaffold engineering, vibration, 3D printing (3DP), system evolution, 3D printed vibratory scaffold

## Abstract

Cell culture and cell scaffold engineering have previously developed in two directions. First can be ‘static into dynamic’, with proven effects that dynamic cultures have benefits over static ones. Researches in this direction have used several mechanical means, like external vibrators or shakers, to approximate the dynamic environments in real tissue, though such approaches could only partly address the issue. Second, can be ‘2D into 3D’, that is, artificially created three-dimensional (3D) passive (also called ‘static’) scaffolds have been utilized for 3D cell culture, helping external culturing conditions mimic real tissue 3D environments in a better way as compared with traditional two-dimensional (2D) culturing. In terms of the fabrication of 3D scaffolds, 3D printing (3DP) has witnessed its high popularity in recent years with ascending applicability, and this tendency might continue to grow along with the rapid development in scaffold engineering. In this review, we first introduce cell culturing, then focus 3D cell culture scaffold, vibration stimulation for dynamic culture, and 3DP technologies fabricating 3D scaffold. Potential interconnection of these realms will be analyzed, as well as the limitations of current 3D scaffold and vibration mechanisms. In the recommendation part, further discussion on future scaffold engineering regarding 3D vibratory scaffold will be addressed, indicating 3DP as a positive bridging technology for future scaffold with integrated and localized vibratory functions.

## 1. Introduction

Traditionally, cultured cells have been grown on treated-polystyrene two-dimensional (2D) surfaces as the standard cell culture plastic-ware. Experiments performed in classical 2D cell culture system have resulted in a large body of knowledge about basic life science [1,2]. However, the morphology of cells that are grown in 2D systems is significantly different to cells in real living tissues, because 2D environments are generally flat, which could only control the growth of cells in x and y directions. In this way, a thorough cell-to-cell interaction will be compromised, which negatively affects protein and gene expression and other cell functions [2,3].

On other hand, cells making up real body tissues usually possess a complex three-dimensional (3D) architecture, which differs remarkably from the flat-monolayer-structure of cells resulted by 2D culture. When considering this, 3D cell culture and its related tools have been developed in recent decades, chiefly for creating suitable 3D surrounding environments that are utilized for optimal cell growth, differentiation, and function [2,3]. The vertical axis of the third dimension tends to be in the range of several micrometers to centimeters, which supports cells to form complex 3D interactions with adjacent cells and generate additional layers [4,5]. System of 3D cell culture also enables cells to develop natural, in vivo-like 3D intercellular interactions, providing an ideal environment for real three-dimensional cell growth and issues, like nutrient exchange, which is similar to intra-capillary exchange in living tissues [1,3,6].

To give an overall picture, dynamicity comes into play on top of 2D and 3D static cell culture. In this connection, the categorization of cell culture can be defined in two ways, one is through its dimensionality and another regards the dynamicity. Knowledge of ‘engineering system evolution’ [7,8,9] can be a good indication to understand this. In general, 2D toward 3D cell culture follows the direction of evolution in dimensionality, and 3D scaffold with 3DP as its popular fabrication tools has played its role in 3D cell culture applications. Dynamic methods on cell culturing, as compared with traditional static means, generally follows the evolution line ‘static to motional or dynamic’, and vibration has been selected frequently as the suitable tool to achieve the dynamicity of cell culture.

## 2. 3D Scaffold Utilized for 3D Cell Culture

As a tendency, 2D cell culture using 2D plates or 2D scaffolds has been gradually replaced by 3D cell culture, and 3D scaffold, as the chief means for 3D cell culture, have been developed.

### 2.1. Definition and Categorization of 3D Scaffold

Term of “3D Scaffold” has been used by previous researches in most literatures. In this review, to have a clearer picture in scaffold engineering, such scaffold, would be defined as “3D static or passive scaffold”, to make a distinction with “3D dynamic or vibratory scaffold” that will be mentioned later in discussion section. 3D scaffolds have specially been developed for 3D cell cultivation. The well-defined and micro-porous architecture provides the native 3D environment where cells can invade, proliferate, and grow [10,11,12]. Two general types of 3D scaffold exist in current tissue and scaffold engineering. First, is usually called 3D cell culture scaffold, which is mainly applied in regenerative medicine or cell behaviour studies; another is 3D tissue engineering (TE) scaffold, which is used for tissue growing and implantation [1,10,13,14,15]. The main difference between them is based on whether the scaffold will dissolve or degrade after use. 3D cell culture scaffold is used in vitro for the purposes of cell cultivation, study, or analysis, and it will not dissolve or degrade after use [6,10,13,16]. TE scaffold is a temporary feature that is used for tissue implantation and it tends to disappear after being implanted to the body. This is achieved through dissolution or degradation. Except the biodegradability issue as chief difference, two types of scaffold can be usually studied together, in terms of their geometric, mechanical, and biomedical issues, and TE scaffold can also be considered as a part of cell culture scaffold, due to the same nature as culturing cells. In this review, these two will therefore be studied and concluded together.

### 2.2. GMB Characterization of 3D Scaffold and Properties

Based on previous literatures regarding 3D scaffold, there are three aspects that generally determine scaffold’s functions, roles, and properties inside cell culture. To help analyse them as one unit, in this review, they will be defined as GMB (Geometric, Mechanical and Biological) characterization for 3D scaffold, representing the geometric, mechanical, and biochemical properties, respectively. In connection with GMB, one vital aspect of scaffold is material composition, which could affect each of GMB and play a significant role for the functionality of scaffold in general. These will be illustrated in this section briefly.

#### 2.2.1. Geometrical Characters

Geometrics have been considered as the one chief important factor affecting the general functions of the 3D scaffold [17,18,19]. Several concepts that are related with geometries and pore, namely pore size, distribution, and association, could participate the definition, classification, and analysis of mainstream 3D scaffold [17,18,20,21]. First, pore size is generally considered as the diameter circumscribing the pores. Porous size of scaffold tends to vary from nano- into micro- levels. Second, 3D scaffold can be defined by its distribution; the numbers of pores per square centimetre vary considerably, generally from around 50 to 600 [22,23,24]. The third category of scaffold is defined by association, which generally means the combination of different shape pores, such as circular and square pores or square and octagonal pores. When it comes to the primal effect of geometrics on cells, geometric control leads to tailored surface topography as well as 3D culturing architectures that help tailored modulation on cell behaviors [15,17,18,25]. In addition, two types of architectures, namely the isotropic or anisotropic ones, have been chiefly applied on the 3D scaffold; both provide specific stimulations on cells as well as various mechanics on scaffold structures [13,17,18].

#### 2.2.2. Mechanics Properties

In addition to geometrics, the mechanics of scaffold are important as they help tissues and cells to maintain the integrities and proper functions. Scaffold’s mechanical properties can be chiefly affected by feature size, structure architecture, and fabrication materials. According to studies [2,13,17,26], physiological processes can be affected significantly by mechanical properties inside cellular environments; these mechanical properties can cause pathological events and affect cells’ behaviors regarding differentiation and growing rate, etc. Mechanical properties of 3D scaffold in general are tissue dependent, with stiffness of tissue ranging from 0.1–3 kPa to intermediate stiffness 8–17 kPa and to higher. Tuning mechanical properties for 3D scaffold design therefore help to ensure tailored physiological stiffness and mechanical supports, which could in turn contribute to better cell attachment in ordinary states as well as better still-vibration alternating mechanism during dynamic cell culture [2,27,28].

#### 2.2.3. Biochemical Controls

When comparing with the properties of geometrics and mechanics on cell culture, as discussed, biochemical properties of 3D scaffold could be considered as the resultant effect from geometrics, mechanics, and the materials composing scaffold. They mainly influence four aspects in cell culturing, that is, cell adhesion, survival rate, proliferation, and differentiation [14,24,28]. Besides this, biochemical control specifically deals with the fictionalization process by biomolecule factors, such as fibronectin and laminin, collagen and polylysine, or growth factors of the Extracellular matrix (ECM) [14,24]. To be specific, one typical effect of biochemical properties concerns short-peptide sequences that can be derived from ECM molecules. These sequences can benefit cell adhesion, like the RGD peptide sequence, as well as increase cell membrane proteins, like E-cadherin [18,24]. 

#### 2.2.4. Material Composition

After illustrating three aspects that determine the functionality of traditional 3D scaffold, another core issue inside scaffold engineering is about scaffold’s material composition. To begin with, the materials that are utilized for 3D cell culture scaffold are generally required to be in-toxic and can easily satisfy food and drug administration (FDA) requirements as well as being biocompatible; the materials utilized for 3D TE scaffold typically need to be bio-absorbable [2,29,30,31]. Previous researchers used to divide materials into biopolymer and non-biopolymer categories, that is, biopolymer materials include natural and synthetic polymers, and non-biopolymer materials chiefly include glass, ceramics, metals, and composites. Varied materials can also be mixed together to reach the required biochemical or mechanical properties of scaffold [2,30,32]. Materials therefore play a vital role in controlling the mechanics and biochemical functions of scaffold, as well as enabling various levels of geometrics in terms of sophistication and complexity. For any kind of dynamic properties that scaffold potentially would be endowed, material selection could also be chiefly important. This will be discussed in a later section.

## 3. Fabrication Methods and 3DP for 3D Scaffold

Following the studies regarding 3D cell culture scaffold and its related characteristics, in the following section, studies toward conventional and 3D printing (3DP) novel fabrication methods for 3D scaffold will be illustrated and summarized.

### 3.1. Conventional Means for Scaffold Fabrication

According to literature studies [2,17,18,33,34], both traditional methods and novel technologies have been used for fabricating 3D scaffold. One common approach to distinguish this could be through judging whether the computer-assisted design (CAD) process is utilized. For conventional technologies that are not utilizing CAD approaches, several methods have been mostly applied, namely Soft Photolithography, Solvent Casting or Particulate Leaching, Phase Separation (TIPS), Microsphere Sintering, Gas Foaming, and Electrospinning. Properties and mechanisms regarding these methods will be given in Table 1.

### 3.2. Concept and Scope of 3DP

As the novel technologies for 3D scaffold fabrication, 3DP or 3D Printing has been used as a term that seems to be ‘universally known’, but different researchers could use it in ways indicating different things. That is, 3DP in many studies failed to give clear idea of what the “3DP” in their context accurately indicate. For example, 3DP was used to refer to a part of solid-free-form (SFF) or rapid prototyping (RP) technologies, that is, the power-based 3DP as the 3D Printing. However, on other researches, for example [30,41,50], 3DP indicated a wider scope containing a range of methods. This could easily cause ambiguity or confusion for following researchers. Therefore, it might always be necessary to define and anchor the “3DP” in solid and crystal-clear way to help readers grasps the exact meaning of this word, as well as understanding the related or derivative term like “3D printed scaffold”. Similar to many concepts in scientific world, two meanings, namely general 3DP and specialized 3DP, based on the indications that are mostly used by previous researchers, can be summarized. For general 3DP, it refers to a range of RP technologies where materials and structures can be additively manufactured in layer-by-layer or drop-by-drop ways, and therefore, it is usually used similarly as Additive Manufacturing (AM). For narrowly defined 3DP, it generally indicates to one small part of the RP or AM technologies that use power and liquid-adhesive. Bearing such information in mind, studies regarding 3DP would follow in a logical and straightforward way. For 3DP and the derivative products as 3D Printed Scaffold, in this review, it indicates to the first class of 3DP. Following this part, novel 3DP methods that are utilized for 3D scaffold will be studied, as follows.

### 3.3. Novel 3DP Methods for 3D Scaffold

**Figure 1:**
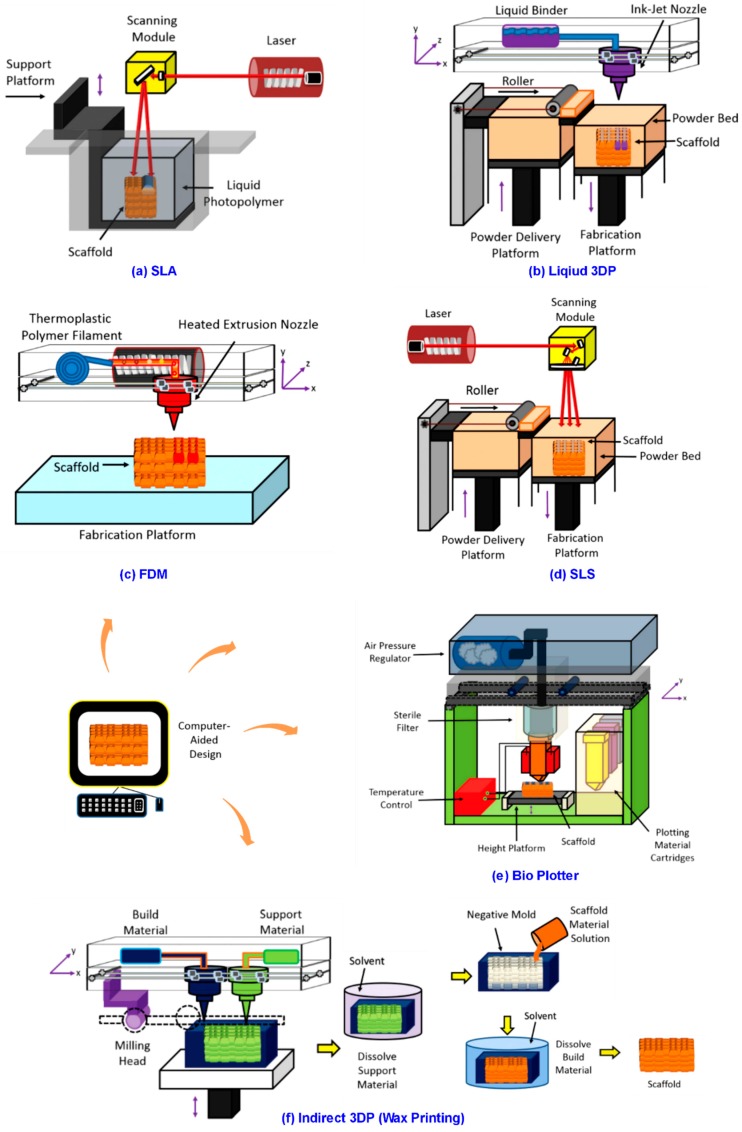
Graphic illustration of six typical three-dimensional printing (3DP) technologies for 3D scaffold fabrication, adapted, and re-structured based on previous work [41] and [53].

Following a clear indication of 3DP, this section will discuss the detailed application of 3DP on 3D cell culture scaffold. Being the novel techniques, 3DP’s high potential ensures that 3DP fabricated scaffolds tend to have finer geometries and material composition when compared with those of traditional ones [18,20,34], and 3DP is predicted to play an increasingly popular role in future’s advanced 3D scaffold engineering. In addition to 3DP, “3D printable or printed scaffold” is to label 3D scaffold that can be fabricated by 3DP methods, making a distinction with scaffolds by conventional technologies. Regarding the systematic classification of 3DP, 3DP technologies utilized for 3D scaffold fabrication chiefly include three systems, that is, 3D laser-based, nozzle-based, and droplet-based 3DP [17,42,51,52]. Table 1 offers a detailed illustration among various 3DP techniques for scaffold fabrication, as well as comparing 3DP with the conventional means. The schematic diagram of several most popular 3DP methods are illustrated in Figure 1. 

#### 3.3.1. 3DP Laser-Based Systems

In 3DP laser-based systems, liquid monomers are photo polymerized or powdered materials are sintered to fabricate 3D scaffolds with complex micro-scale structures [2,33,54]. Stereolithography (SLA) is one typical technology and it is utilized to fabricate products, like bone-like 3D scaffolds [55]. It consists in a photo-polymerization using UV laser to build layered structures. A liquid photo-curable monomer resin polymerize when exposed to UV light. The laser scans the top of a bath, polymerizes the resin, and creates a solid layer. For sequent layers, the same process is repeated by moving down the platform. A post-process treatment is to cure the scaffold in UV oven. SLA allows the fabrication with complex and anatomically shaped structures [43]. Besides SLA, two-photon polymerization (2PP), where Femto-second laser beams are focused on the photosensitive liquid material that is mixed by monomeric matrix molecule and a photo initiator, is another technology. Two photons are simultaneously absorbed, causing high peak energy in the focal volume of the focused laser beam. Energy exceeding a threshold, the photo initiator molecules will be excited; resulting in a highly localized chemical polymerization event that is confined to laser’s focal volume [2,17,18,33,54].

#### 3.3.2. 3DP Nozzle-Based Systems

In this category, ink materials can be accurately dispensed through a nozzle following the filament-on-demand principle by fused deposition modeling (FDM) or drop-on-demand principle by 3D plotters. Nozzle treats the materials chemically or thermally. After processing, the ink solidifies via gelation or solvent-induced phase changes. The mobile nozzle determines the X-, Y-resolution, and Z- is controlled by platform [17,33,56]. Among nozzle-based systems, FDM is the most popular one. The nozzle pushes out a thermoplastic polymer filament and it dispenses the melted polymer onto a platform by a layered-process. It has been used to manufacture 3D scaffold of honeycomb-like pattern with fully interconnected porous network, and FDM is also a solvent free method. However, it is a necessity to prepare polymeric filaments before fabricating. This makes the FDM procedure longer and expensive. The narrow range of materials makes FDM a harder to achieve scaffold with complex filament composition and the fixed diameter of filament and needle tip also limits the printable resolution [17,33,35].

#### 3.3.3. 3DP Droplet-Based Systems

Droplet-based 3DP can produce complex independent 3D patterns with multiple structural constituents and properties. The highly defined control of macroscopic features can affect the growth rate of cells inside fabricated scaffold, which can be the unique advantage of this system. Droplet-based systems are sometimes conceptually similar to selective laser sintering, which means that powdered starting materials are sequentially and selectively solidified to form 3D structures layer-by-layer. The difference is in the use of liquid chemical binders rather than laser light to solidify the material [25,33,56]. 3DP droplet-based techniques mainly include ink-jet printing, direct-write printing, and micro pen writing [17,33,35]. For precise deposition, this system can utilize different materials, such as wax, liquid biomaterials, or a chemical binder onto a surface to construct 3D scaffold with specific chemical properties [25,33,49].

After illustrating novel 3DP technologies and traditional methods for scaffold fabrication, another benefit of 3DP could be addressed here as well. That is, 3DP methods have shown more applicability for fabricating dynamic or vibratory structures on application of scaffold as well as other biomedical things. Potential vibratory or active shape-changing structures that are based on materials, such as shape-memory polymers (SMPs) and shape memory nanocomposites (SMNCs), are able to be controlled in both space and time and gaining ascending attention in scaffold engineering. However, conventional fabricating approaches, for example, the methods that are mentioned above, have shown less design applicability on such smart or dynamic structures [57,58]. In contrast, 3DP methods, in either of the three systems as studied, have shown their fabricability in effectively achieving active shape-changing structures [57,58,59]. This means that, despite the role of 3DP to help cell culture scaffold evolve from 2D into 3D, it also benefits the direction of scaffold evolving from static into dynamic. How to further utilize this benefit of 3DP inside scaffold engineering, however, is still under ongoing research.

## 4. Vibration Mechanisms Applied for Cell Cultivation

In previous sections, knowledge regarding 2D into 3D cell culture, 3D cell culture scaffold, and 3DP fabrication methods have been studied. The direction of this can be concluded as the dimensional evolution in cell culture and scaffold engineering, where 3DP can be seen as the positive force helping to direct this evolution. On hand other, another line of evolution regarding cell culture and scaffold also needs to be considered, that is, cell culture with its related platforms tend to evolve from static into dynamic. There are several reasons for this, and vibration mechanisms have been mostly utilized as tools for achieving this development.

### 4.1. Vibration and Dynamicity

When comparing with the roles that are played by 3D scaffold, which is for creating in vivo 3D surrounding environments for issues, like optimal cell growth, differentiation, and other cell functions, vibration stimulation firstly aims to provide a dynamic or active environment that can be similar to the in vivo conditions, where cells tend to grow in better and natural way, including increased cell adhesion and better differentiation rate, etc. [60,61]. Besides this, when considering the benefits from mechanical stimulation on cell culture, such as increasing cell growth rate [62,63], and that cells that are cultured in current 2D or 3D artificial environments are normally free from such stimulations, researchers have attempted to develop methods for creating dynamically stimulating conditions. Vibration mechanisms have been used for this purpose [60,64,65]. The third beneficial effect of utilizing vibration can be that cells can be cultured in tailored dynamic conditions where researchers can study various aspects of cells based on specific biomedical needs [60,66]. For example, the intensity of vibration can be controlled in ascending or descending levels to help analyse the difference of cell proliferation in natural in vivo states. The application of vibration mechanism therefore has been vitally important, in terms of mimicking real dynamic environment in vivo, creating necessary mechanical stimulus as well as enabling adjustable intensity and evaluating dynamic cell studies.

Out of several factors regards vibration, generally including frequency, amplitude, wave length and flexibility in vibration control etc., vibration frequency with its related properties such as frequency range and intensity could be the most important factors when it comes with vibration in cell culture. Vibration with different frequencies, for instance, can easily have different effects on issues, including cell lifecycles, proliferation rates, and alkaline phosphatase (ALP) activities, etc. [67,68]. In terms of the vibration frequency range, cells can be responsive to the broaden frequency vibrating strain and can also be sensitized to sinusoidal stimulation under such strain [65,68,69]. Continuously changing frequency under a certain range can affect the rate of cell differentiation, and vibration with tailored frequency range can help cells to survive relatively longer when compared with traditional static culture [64,68,70,71].

### 4.2. Vibration Systems Utilized for Cell Culture

After discussing the vibration regarding dynamicity in cell culture, in this section, we will introduce several mechanisms or methods that have been mostly applied to achieve vibration in cell culture applications. Several things need to bear in mind in first place. Based on the literature studies from precious decades, it seems that the development from 2D into 3D cell culture, has witnessed a rapider rate when comparing with that from static into dynamic culture. Advanced 3D scaffold with increasingly complex geometries and material composition is becoming popular gradually, whereas vibration mechanisms rarely can be seen regarding the recent development or novel application on cell culture studies. The reason of this might partly lie on the fact that rapid development of 3DP, which accelerates the evolution of 3D cell culture, and partly due to that previous researches might tend to attribute more significance in 3D scaffold regarding 3D culture than developing or studying novel vibration mechanisms, which would benefit the dynamicity of cell culture. Taking these into consideration, it becomes reasonable why many previous studies introducing vibrations on cell culture tend to use 2D culturing plates or early-stage 3D scaffolds with very simple inner structure and material composition. In following part, several types of vibration stimulation previously used as mainstream vibration means for dynamic cell culture will be introduced and studied. Table 2 will compare and summarize these previously utilized vibration systems.

#### 4.2.1. Bioreactor-Based Vibration System

In many cases, bioreactor-based systems have been used to provide vibration on the cultivation of cells in bone and cartilage applications [72]. Bioreactor generally provides a continuous or time-controllable inner vibration for cells and culturing platform or scaffold that would be put inside the bioreactor [86]. Recent bioreactor systems tend to be utilized for several purposes, which includes measuring the oxygen content, culturing anatomically shaped grafts, developing perivascular network, as well as evaluating the relationships between scaffold environment and the signalling pathways [73,87]. Besides this, the application of bioreactors also creates the possibility for scientists to study the dynamic factors of cells, such as the oxygen contents, issues regarding shear, and stem cell differentiations [74,75]. Among these factors, the increase of cell differentiation could usually be considered as the primal benefit from bioreactors.

#### 4.2.2. Loudspeaker-Based Vibration System

Previously, a large number of dynamic systems applying mechanical stimuli on cell culture have been designed to simulate aspects, like stretch, compression, or shear stress, etc., but not on vibration [88]. To fill this gap, a loudspeaker-based system has been invented to study cells behaviours under cellular vibratory conditions. In one previous research that can be considered as typical, a device driven by a sinusoidal signal at 60 Hz with a power amplifier was used to subject cultured cells into vibratory conditions. The main aim of study was to test whether vibration could modify cell proliferation, and the experimental result showed a ‘yes’ answer with the proof that the concentration of interleukin-8 (IL-8) had increased.

#### 4.2.3. Vibration System from Mechanical Stimulators

Several researches have utilized mechanical stimulators on dynamic cell culture, where means like piezoelectric actuator or vibratory transducer have been commonly applied for generating vibratory stimulations [65,79,89]. Bone cells, like osteoblasts, have been frequently studied using such stimulators to compare with cells that are cultured in static. To be specific, the effects of broad frequencies with low amplitude strains on bone and hESC cells have been studied. Results showed that osteoblasts can respond to a broader range of frequency strain from mechanical stimulator, cells tend to be sensitized to sinusoidal stimulations, and a higher number of cell proliferation was resulted after period of weeks [79,80,81]. In brief, mechanical stimulators could affect the differentiation and it implies the existence of a frequency-dependent effect from vibrations.

#### 4.2.4. Vibration System from Ultrasonic Generators

After mechanical stimulation with relatively low frequency having been applied on cell cultivation, vibration from ultrasonic system was applied aiming to break the low-frequency mould and open avenue for vibration stimulation with higher frequencies [90]. Relevant experiments have studied how cells proliferate, grow, and differentiate under continuous or discontinuous ultrasonic vibration. The prominent effect could due to the created macroscopic flow, generating a stirring or heating effect, which in turn changes cell behaviors. It has been pointed out that low frequency vibration makes flow in the liquid, while vibration with high energy could have the possibility to dismantle existing structures of cells. It has also indicated that while mild vibration can potentially accelerate cells’ proliferation and differentiation, the optimum intensity of such vibration has to depend on specific kind of cells [82].

#### 4.2.5. Vibration System from 3D Micro-Vibration Stages

3D micro-vibration stage has been developed as a novel system generating tailored vibrations on each direction, which helps to exert vibratory stimulation onto cultured cells in a non-invasive and 3D manner. Its effects being investigated morphologically, experimental result shows that such vibration stimulation can decrease the projected area and increase the slenderness ratio [83]. Based on the slenderness ratio result, the vibration stimulation makes value of slenderness ratio larger. Given that cellular senescence usually makes value of slenderness ratio smaller, the results may be the evidence that such vibration stimulation could generate a beneficial effect on gene expression by affecting the gene expression pattern and makes the cells remain in a genetically younger state.

#### 4.2.6. Vibration System from Mechanical Micro-Vibrators

Biomedical researches [81,84,85] have proven that in vivo there exists multiple mechanical forces, the combination of which can induce cell-to-cell communication, which exerts beneficial effects by refreshing the fluid surrounding cells and eliminate metabolites. Studies to achieve higher quality embryos by external stimuli using machines to replicate in vivo mechanical forces have been reported recently, and following this trend, systems of mechanical micro-vibrator have been developed as generating external stimulus. This micro-vibration stimulus has been experimented in cells culture of mouse embryonic, showing the beneficial effect on general cell development, with similar beneficial effects existing regarding pregnancy and implantation rates to the human embryonic culture [84].

It is worth noting that the categorization of ‘system’ in this research is chiefly based on two aspects, first the strategies or devices that are utilized for generating vibration, and second, the purposes to apply such vibration on cell culture applications. For knowing the details of vibration device or working components, readers can refer to the corresponding literatures listed.

## 5. Discussion

In previous sections, we have studied 3D cell culture scaffolds, 3DP methods, as well as vibration mechanisms that are applied for cell applications. In this section, the limitations and gaps regarding these would be analyzed, which could help to better understand the current situations as well as giving potential recommendations for future.

### 5.1. Current Limitations and Gaps

Several limitations might need to be addressed. One limitation of the currently and previously applied vibration mechanism is that the vibration that is required in cell culture can only be achieved by outer mechanical stimulations; bio-reactors, mechanical stimulators, and vibration stage, etc. are the popular ways to generate vibrations in cell culture in vitro. In many cases, cells have to be limited in receiving vibrations through connecting culturing platforms to external vibrators or mechanical shaker vibrates [60,65]. Several defects may exist. Researchers first of all find it difficult to accurately control the cell-received vibratory frequencies. Reasons can be that there exists three ‘layers’ regarding the whole cell culture unit, namely external vibrators, 2D or 3D cell culture scaffold, and the cells cultured inside. Inside real tissue, however, there is the all-in-one dynamic organism, which ensures that cells are cultured in an exact and thorough way, as required by the organism. Utilizing externally applied vibratory mechanism cells are very likely to be affected by these intermediate “layers”. For example, vibrations usually have to pass through the vibrators or vibration devices, platform holding or containing the vibrators, scaffold inside or on top of platform, and the inner environment of scaffold, after which it would finally reach cells [67,70,89,91]. The vibration properties such as frequency generated externally and received by cells therefore might be unidentical or undesirable, which could compromise the precisely-controlled vibration. In addition to this limitation, another gap might be that current vibration mechanisms could merely achieve the vibratory effects that would be ‘evenly-distributed’ among different areas inside the scaffold. Logic of this is simple, that is, when external vibration works, all parts of culturing scaffold have to react together passively. This negatively affects some differentiation and proliferation of cells into 3D tissues when different group of cells need a tailored and variable dynamic sub-environment [92,93]. For instance, organs or tissues in vivo, like bone or lung, usually can be divided into different sub organ or tissue group where stems cells will specifically develop into, and distinct parts may therefore require different or specific vibratory stimulus or dynamic properties when comparing with others [64,68,93,94,95]. This gives a good indication that vibration stimulation on cell culture scaffold may need to be capable of providing localized and tailored vibrations inside different sub areas of scaffold itself, in order to help cells to grow or develop into tissues at the required way.

After analyzing the gaps of currently applied vibrations, limitations of traditional 3D scaffolds could follow in an easy and logical way. To be specific, 3D scaffolds, as discussed, have remained passive or static in general and they could not generate dynamic stimulations directly to cells, which will potentially shorten the ‘passing layers’ discussed above. On other hand, previous studies have chiefly focused on developing or optimizing the passive parameters, including scaffold’s chemical composition, mechanical property, and geometry [18,60,94]. For example, rarely could we find relevant resources, perspectives, or discussion regarding potential “self-vibratory scaffold” or ‘scaffold generating localized vibrations’, and general innovation works toward such scaffold product could not be found. The lack of focus and attention of current researches on such direction thus might be considered as another limitation, hindering the further development of 3D scaffold regarding better and advanced vibration mechanisms on cell culture applications.

### 5.2. Future Trends and 3D Vibratory Scaffold

After discussing the current limitations and gaps, in this section, we will discuss the future trend regarding aspects on cell culture, scaffold engineering, vibrations, and 3DP.

#### 5.2.1. Trends Regards Cell Culture Dimensionality and Dynamicity

**Figure 2:**
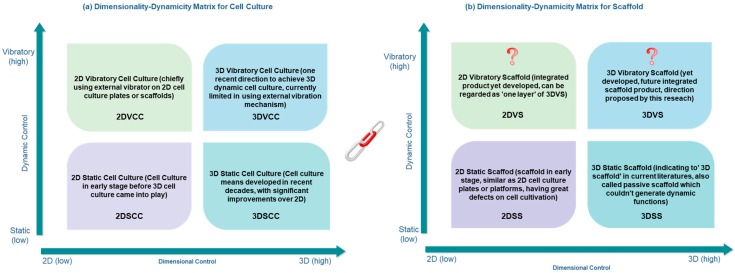
A graphic illustration regarding (**a**) four main stages in cell culture development, and (**b**) development aspects in terms of scaffold engineering for cell culture applications.

Parallel with the issues of dimensionality in cell culture, dynamicity will be another vital concept to categorize cell culture systems. Despite being either 2D or 3D, cell culture based on dynamicity can be further divided into static culture and dynamic culture, and vibration mechanism has been used as chief tool to achieve the dynamic purposes. Detailed knowledge has been addressed in the vibration section, and here in Figure 2 we briefly summarize the four categorization of cell culture, as well as the development vision of scaffold engineering. This diagram might also indicate general knowledge regarding the system evolution trends from 2D culture into 3D, and from static into dynamic.

Based on literature studies, the previous evolution line of cell culture and scaffold engineering can be concluded as: 2D culture using 2D static or passive plates has largely evolved into 3D culture utilizing 3D scaffold, and static cell culture has partly evolved into dynamic cell culture with several vibration mechanisms being applied. In terms of the research scope on cell culture scaffold, previous works can be considered as thorough to go through 2D static, 3D static, and 2D dynamic stages, while the exploration, attention, and studies regarding 3D dynamic still remains at an early stage and much work is yet started.

Following the visual illustration as in Figure 2a, we know that current focus includes four aspects of cell culture, where 3D dynamic cell culture (3DDCC) has been the chief focus in modern research. For scaffold development in Figure 2b, it started from 2DSS and currently stays at 3DSS. Because of the higher usefulness of 3D methods than 2D, 2DVS is less probably to be developed as a product. Indeed, regarding it as one layer of the 3DVS might be more appropriate. Herein, 3DVS can be the product in following scaffold engineering. To conclude, it is logical to predict that 3DDCC will continuously be the chief focus for current and future cell culture, and 3D Vibratory Scaffold (3DVS) will be most likely developed to firstly play general roles cooperating with 3DDCC, and secondly mitigating the gaps and limitations discussed previously.

#### 5.2.2. 3D Vibratory Scaffold in Future

The concept of the future vibration-integrated scaffold product, which would play a significant role in 3DDCC, might be temporarily named as “3D Vibratory Scaffold”, where separate mechanisms of vibration and scaffold would turn into unified systematic functioning with some required vibratory functions would be attributed inside the scaffold itself. There are several indications why 3D static or passive scaffold, in next stage of scaffold engineering, would most likely evolve into 3D vibratory scaffold. Laws for general system evolution [7,8,9,96], in the first place, indicate that separate elements of multiple systems generally evolve into the single integrated system with multiple functions. Besides this, when considering the urgency and necessity to address current limitations or gaps as discussed, developing better vibration mechanisms and 3D scaffold that could utilize such scaffold-vibration integration would be of significant value, which makes best of both worlds in positive effects from vibration and 3D scaffold on cell culturing. Scaffolds therefore could potentially generate tailored and localized vibration and frequencies with higher cell culture accuracy and controllability.

Two chief future trends therefore would follow. First, the combined unit of vibration mechanism and 3D passive or static scaffold on cell culture has great potential to be developed. In the near future, 3D passive scaffold could have the high possibility to evolve into 3D vibratory scaffold. Second, several evolution laws support and indicate the direction of the scaffold-vibration unification; that is, the passive evolves into the active, the separated into the integrated, and the single-functional into the multi-functional. Following these two trends, alone with the increasing call from the scientific world toward limiting biomedical testing on living animals [97], the development of potential 3D Vibratory Scaffold, which better mimics real in vivo conditions could be partly used as an alternative for medical testing in animals, which can be considered as the third trend. In addition to these, the advanced 3D printing (3DP) methods will possibly witness higher applicability as the tendency in future scaffold engineering [37,98,99]. 3DP will potentially be utilized as fabrication method for both advanced/novel 3D static and passive scaffold, but also the 3D vibratory scaffold that could emerge in near future, the discussion of which will be illustrated in following section.

#### 5.2.3. 3DP as Bridging Technology for Future Scaffold

Future scaffold in the next development circle, as discussed, might integrate vibratory functions into scaffold itself, and the fabrication methods will therefore be one vital aspect to be reflected upon when judging whether the designed or proposed scaffold is practically achievable. Among different technologies for traditional scaffold fabrication, 3DP can be predicted as probably the best solution for fabricating the future 3D vibratory scaffold (3DVS), which has been pointed out as future trend of scaffold engineering in this review studies. Several evidences could support this prediction.

First of all, since 2DVS can be considered as one layer of 3DVS, as illustrated, designing 2DVS first then fabricating it in layer-by-layer way to constitute 3DVS would be a highly useful approach. 3DP technologies has the exact layer-based CAD approach, and makes it probably the optimal tool for achieving 3DVS. Following this, the current and continuously increasing advances in 3DP associated with tomographic reconstruction and intellectual modelling could allow for current complex scaffold architectures with a further range of length scales, as well as higher geometric options [37,100]. This could evidentially benefit aspects, such as design flexibility and structural fabricability in future 3D scaffolds when focusing on potential design requirements of 3D vibratory scaffold. In addition, feasible with a wider range of 3DP materials that are equipped with electrical, optical properties and dynamic or magnetic properties [59,98,99,101,102], material composition concerns for 3D vibratory scaffold might benefit most from 3DP technologies when compared with other fabrication tools. As new biocompatible materials and “bio-inks” being created or synthesized [35,103,104], 3D printed scaffolds used in tissue and cell engineering tend to become more effective in cell culture applications, and this helps to cooperate with future scaffolds, which tend to have the same application as cultivating cells. Furthermore, hybrid 3DP approaches, alone with novel 3DP methods gradually being discovered [37,105,106,107], also make the 3D scaffold in next generation more promising. These hybrid systems could have the potential to mitigate the disadvantages of any 3DP technology used alone, such as the limited material selection of definite 3DP. Last, in advanced cell-medical applications, the potential future scaffold might need the fabrication mode to be of small quantity, but of high quality and rapid in time [31,98,106]. This is different from fabricating other daily engineering products where fabrication costs and output quantities would be generally considered in priority.

In brief, as current 3DP techniques are further fine-tailored and more bio-functional materials become available, the design of 3D vibratory scaffold could be partly due to how to make best of the 3DP characteristics inside scaffold itself. Studying 3DP techniques and related properties regarding dynamic materials may contribute to part of design solutions, which enables scaffold generating tailored vibrations. Therefore, the potential future 3D scaffold product, being both ‘vibratory’ and ‘3D printable’, as discussed, could be achieved by novel 3DP technologies, which might probably contribute a new term in future scaffold engineering world, namely ‘3D printed vibratory scaffold‘ (3DPVS).

## 6. Conclusions

This review paper explained the evolution line from static cell culture to dynamic cell culture and from two-dimensional cell culture to three-dimensional cell culture. A summarizing table concluded the state-of-the-art of 3DP technologies for 3D cell scaffold fabrication. As to the dynamic cell culture discussion, different vibration methods that were previously applied on cell culture were reviewed. Further discussion on the future trend of dynamic 3D cell culture and 3D vibratory scaffold was addressed. With the high feasibility and wide material selectivity, 3DP might be a good bridging technology for future scaffold with integrated local vibratory functions.

## Figures and Tables

**Table 1:** Comparison table of various 3DP technologies for fabrication of three-dimensional (3D) cell scaffold.

3DP Methods	Chief Feature & Mechanism	Materials	Cells Studied	Architecture	Dynamic Structure Appli-Cability	Advantages	Disadvantages	Refs.
**Two-Photon polymerization (2PP)**	Laser beam is focused onto a liquid material; CAD	Solidifable fluid: photosensitive materials	Bone cells, human stem cells	Mesh-like, wheel-, pyramid-, cube-like pattern in hydrogel	High	Homogeneous and two-composite polymer	Excess of initially powdered material hard to remove	[2,35,36]
**Laser Engineered Net Shaping (LENS)**	Metal powders used to build or repair scaffold parts	Fine powder: plastic, metal etc.	General tissue cells	Mesh-like network	High	Able to repair old parts and fabricate new; secondary firing process not needed; excellent material properties	Low geometrical control in dimension	[18,37,38,39]
**Stereolith-ography (SLA)**	Laser onto liquid photopolymer to generate scaffold; CAD	Solidifable fluid: photopolymer resins, temperature sensitive polymers, ion cross-linkable hydrogels, ceramic paste, etc.	Rat bone, rabbit trachea, pig tendon cells	Mesh-like, Honeycomb- Wheel-, pyramid-, cube-like; porous cylinder	High	High surface quality, high resolution, high complexity, fast speed.	Limited to specific polymers (photopolymers); need support system; moderate strength; expensive	[36,40,41,42,43,44]
**Selective Laser Melting (SLM)**	Using small diameter wire-frame elements	Fine powder: Plastic, metal, ceramic or composite powders	Mouse bone cells	Mesh-like, Honeycomb- Wheel-, pyramid-, cube-like network	High	Controlled pore interconnectivity and porosity; greater durability of mould; free from temperature-related defects	Low surface quality	[35,40,45]
**Selective Layer Sintering (SLS)**	Laser-based CAD technique; include laser and power bed	Fine powder: Plastic, metal, ceramic or composite powders	Mouse bone, rat heart, rat bone, mouse skin, mouse heart cells	Mesh-like network, porous cylinder	High	Good mechanical strength; complex structures; high resolution; large part size; no support structure needed	High materials requirements (heat, shrinkage resistant); require high processing temperature; powdery surface; costly; time consuming	[2,40,41,42]
**Laminated Object Manufacturing (LOM)**	layers of adhesive-coated laminates being successively glued together and cut to shape with laser	Laminated thin sheet: Ceramics—alumina, silicon nitride, and zirconia and metals	General tissue cells	Mesh-like network	High	Large part size; layer builds quickly; fine accuracy and resolution low cost	Materials limited	[21,40,46]
**Ink-jet Printing (3DP in traditional terminology)**	Liquid binder jetting; drop-on-powder; CAD	Hydroxyapatite, magnesium phosphate, cement, polyurethane	Rat bone, rabbit bone and mouse bone cells	Mesh-like network; porous cylinder	High	Materials versatile; powder can be trapped inside body; don’t need support structure; high speed; cost-efficient	May be toxic; low mechanical strength compared with Laser printing; time consuming in post-processing	[2,21,28,41,42]
**Fused Deposition Modeling (FDM)**	Thermoplastic polymer through heated extrusion Nozzle to create scaffold onto platform; CAD	Non-brittle flament: Thermoplastics like ABS, PLA, and PCL etc.	Rat and Swine Bone cells	Mesh-like network; porous cylinder	High	Relatively inexpensive; low cytotoxicity; good strength; no support structure needed; no power trapped; good mechanical anisotropy; speed control by strand diameter	Limitation on materials (thermoplastics); materials non-biodegradable; support structure required for complex geometrics; post possessing needed; low resolution; low speed	[2,21,28,41,42]
**3D Plotting (Bioplotter Printing)**	Air pressured system to extrude material from bioink cartridges	Solidifable fluid: ion cross-linkable hydrogels etc.	Rabbit cartilage, rabbit trachea, rat cartilage, mouse cartilage, mouse skin cells etc.	Mesh-like network; dot-like structure	High	Viable cells printable; soft tissue applications; wide variety of natural and synthetic materials; processing at room temperature	Nozzle may be cytotoxic; support structure required when printing complex structure; low dimensional accuracy	[22,28,40]
**Wax Printing (Indirect 3DP)**	Wax being printed as a negative mold where scaffold solution is cast	Wax	Rat bone cells, mouse stem cells	Mesh-like structure	High	Benefit on preproduction; versatility on material casting following obtained mold	Materials may fail to be biocompatible; Low resolution; always need a mold; low speed in fabrication	[41,45]
**Conventional Methods**	**Chief Feature & Mechanism**	**Materials**	**Cell Studied**	**Architecture**	**Dynamic Structure Appli-Cability**	**Advantages**	**Disadvantages**	**Refs.**
**Electrospinning**	Polymer solution forced into a capillary to form a jet of solution a tip; high voltage applied between tip and collector	Biodegradable polymers like PCL	Rat bone, mouse bone, rabbit vascular tissue cells	Mesh-like structure; microchannel	Low	Fast speed; cell printing available; soft tissue application; similar to ECM; better mechanical control (shear stress); high aspect ratio and surface area	Fibers printed in random orientation; pore sizes not uniform; high voltage demand; organic solvent needed	[2,41,42]
**Solvent Casting/Particulate Leaching**	Dissolute polymer in an organic solvent and casting into a mould	Composite like PLA/Calcium phosphate	Bone cells	Mesh-like structure	Low	High geometric control; easy processing; fast speed	Organic solvents have to be used	[42,47]
**Phase Separation**	Polymer and solvent mixed pass through a freeze-dryer	Ceramics, i.e., glass	Bone osteoblast cells	Homogeneous and highly porous structures	Low	High porosity; easy to cooperate with other techniques	Possible shrinkage issues; organic solvents used; anisotropic pores	[42,45,48]
**Gas Forming**	Using a process with high-pressure carbon dioxide at room temperature	Polyesters polymers; biodegradable polymers	Bone cells	Mesh-like; microchannel	Low	Organic solvents not needed; room temperature processing; macro-porous scaffold	Poor geometrical and porous control	[23,42,45]
**Microsphere Sintering**	Sintering polymer microspheres thermally or chemically	Polymers	Bone cells	Mesh-like; microchannel	Low	Pore size being gradient; complex shape fabricable	Lack of control in interconnectivity	[42,45,49]

Note: Green represents 3DP laser-based technologies, orange for droplet- or powder-based and yellow for nozzle-based ones. Grey colour represents traditional tools for scaffold fabrication.

**Table 2:** Previously applied vibration mechanisms on cell cultivation and illustration of their functions and properties.

Vibration System	Devices Applied	Purpose of System	Scaffold Applicability	Vibration Properties/Frequency	Cells Applications	Effects on cell Culture	Unique Strengths	Limitations	References
Bio-reactor System	A device, like a vessel or container, where cell culturing is carried out	Study the dynamic factors of cells, including oxygen contents, shear, differentiations	Yes, both 2D and 3D	Most frequency 10–200 Hz; amplitude 0–5 mm etc.	bone and cartilage cells, MSCs cells etc.	Increased proliferation; help gene expression etc.; increased cell viability	Tend to be inexpensive, easily establishable	Frequency cannot be precisely controlled	[72,73,74,75,76,77]
Loudspeaker-based Vibratory System	A subwoofer loudspeaker, water-proof Mylar speaker etc.	In vitro platform for evaluating cellular responses to vibration	Yes, chiefly for 2D	Frequency 60–1600 Hz, amplitude 0–30 mm etc.	MSCs cells, vocal fold cells	Help proliferation, help release some cell product, like IL-8	Relatively accurate and stable	Extra tools needed to calibrate the System; limited in cell application	[60,77,78]
Mechanical Stimulator System	External device, like piezoelectric actuator or vibratory transducer	Investigate the frequency-dependent effect from vibration	Yes, both 2D and 3D	Frequency 30–200 Hz, amplitude 0–30 mm etc.	Majorly in Bone cells, osteoblasts	Benefit gene expression, proliferation and differentiation	Easily accessible, and widely applied	Limited cell application; inflexibility of frequency control	[79,80,81]
Ultrasonic vibration System	Piezoelectric element, Ultrasonic generator etc.	Study cell behavior under vibration stimulation with higher frequencies	Yes, both 2D and 3D	Frequency 100 Hz–1 MHz, amplitude 5–50 μm etc.	Myoblast cells etc.	Increase the proliferation of cells	Capability of generating high frequency	May damage cells and hinder normal proliferation	[69,82]
3D Micro-vibration Stage	A micro-vibrator stage basically consists embedded vibrator	Study the cell behaviors in dynamic culture morphologically	Yes, chiefly for 3D	Frequency 10–50 Hz, amplitude 30–50 μm	human osteoblast cells etc.	Non-invasive and three-dimensional vibration	Affects gene expression pattern and makes the cells remain younger	Limited frequency range; May damage cells	[60,83]
Mechanical Micro-vibrator System	A micro-vibrator electric device	Mimic dynamically mechanical forces in vivo, evaluate vibration responses	Yes, both 2D and 3D	Frequency 10–100 Hz, amplitude 0–5 mm	mouse and human embryo etc.	Precious frequency and time control	Benefits cell’s in vitro fertilization and development rates	Limited frequency range; special device needed	[84,85]
